# Prevalence, Distributions and Determinants of Obesity and Central Obesity in the Southern Cone of America

**DOI:** 10.1371/journal.pone.0163727

**Published:** 2016-10-14

**Authors:** Fernando Lanas, Lydia Bazzano, Adolfo Rubinstein, Matias Calandrelli, Chung-Shiuan Chen, Natalia Elorriaga, Laura Gutierrez, Jose A. Manfredi, Pamela Seron, Nora Mores, Rosana Poggio, Jacqueline Ponzo, Hector Olivera, Jiang He, Vilma E. Irazola

**Affiliations:** 1 CIGES. Universidad de La Frontera (UFRO), Temuco, Chile; 2 Department of Epidemiology, Tulane School of Public Health and Tropical Medicine, Tulane University, New Orleans, Louisiana, United States of America; 3 Instituto de Efectividad Clínica y Sanitaria (IECS), Centro de Excelencia en Salud Cardiovascular para el Cono Sur (CESCAS), Buenos Aires, Argentina; 4 Sanatorio San Carlos, Bariloche, Pcia de Río Negro, Argentina; 5 Departamento de Medicina Familiar, Universidad de la República, Montevideo, Uruguay; 6 Municipalidad de Marcos Paz, Pcia de Buenos Aires, Argentina; Indiana University Richard M Fairbanks School of Public Health, UNITED STATES

## Abstract

**Background:**

Obesity is a major determinant of cardiovascular disease in South America. However, population-based data are limited.

**Methods:**

A total of 7,524 women and men, aged 35 to 74 years old, were randomly selected from 4 cities in the Southern Cone of Latin America between February 2010 and December 2011. Obesity clinical measurements and cardiovascular risk factors were measured using standard methodology.

**Results:**

The prevalence of obesity and central obesity were 35.7% and 52.9%, respectively. The prevalence of obesity and central obesity were higher in women, and even higher in women with lower education compared with women with higher education. In men and women obesity was associated with a higher prevalence of diabetes, odds ratio (OR) 2.38 (95% Confidence Interval [CI]: 1.86 to 3.05) and 3.01 (95%CI 2.42 to 3.74) respectively, hypertension (OR 2.79 (95%CI 2.32 to 3.36) and 2.40 (95%CI 2.05 to 2.80) respectively, dyslipidemia (OR 1.83 (95%CI 1.50 to 2.24) and 1.69 (95%CI 1.45 to 1.98), respectively, low physical activity (OR 1.38(95%CI 1.14 to 1.68) and 1.38 (95%CI 1.18 to 1.62) respectively and a lower prevalence of smoking (OR, 0.65 (95%CI 0.53 to 0.80) and 0.58(95%CI 0.48 to 0.70) respectively.

**Conclusions:**

Obesity and central obesity are highly prevalent in the general population in the Southern Cone of Latin America and are strongly associated with cardiovascular risk factor prevalence. These data suggest that efforts toward prevention, treatment, and control of obesity should be a public health priority in the Southern Cone of Latin America.

## Introduction

Cardiovascular diseases (CVD) are the leading causes of death in the Americas, accounting for 33.7% of total mortality [1]. Coronary heart disease (CHD) and stroke are the two most common forms of CVD and the leading causes of deaths in Latin America [2]. It is estimated that CVD mortality in Latin America will increase by more than 60% between 2000 and 2020, compared to an increase of only 5% in the developed world [3]. It is highly anticipated that this trend will continue due to population aging and the adoption of Western lifestyles that lead to increased sedentary behavior and obesity. These factors in turn contribute to increased rates of hypertension, dyslipidemia, and diabetes mellitus (DM) [4].

Over recent decades, obesity has become a global epidemic, representing a major cause of disability and mortality [5,6]. Although obesity is associated with other downstream cardiovascular risk factors, such as DM and hypertension, it is also an independent risk factor for CVD [7]. In the United States, an obesity prevalence of 68.5% has been reported in adults >20 years old [8]. Population growth and social development in Latin America has led to a demographic transition, increasing the frequency of obesity occurrence, with alarming prevalence reported in some countries in the region [9–16]. In several countries, a pattern of obesity occurring more frequently in both lower socioeconomic levels and lower levels of education is observed repeatedly [12,14,15].

In a case-control study of 1,237 CHD patients and 1,888 controls in South America, central obesity was associated with highest population-attributable risk at 48.5% [17]. However, population-based data on the prevalence of these conditions are sparse in Latin America. The CESCAS (Centro de Excelencia en Salud Cardiovascular para America del Sur) I is a population-based cohort study examining CVD and risk factors in a representative sample of the general population of the Southern Cone of Latin America [18,19]. The objectives of the present analysis are as follows: (1) to estimate the prevalence of obesity, overweight and central obesity in the Southern Cone of Latin America; and (2) to assess the association of obesity with CVD risk factors in the general adult population in the Southern Cone of Latin America.

## Material and Methods

### Study participants

The details of study design and sampling method of the CESCAS I study have been published earlier [18, 19]. Briefly, 7,524 women and men, aged 35 to 74 years old, were recruited between February 2010 and December 2011 from randomly selected samples in 4 cities in the Southern Cone of Latin America: two cities located in Argentina (Bariloche and Marcos Paz), one in Chile (Temuco), and one in Uruguay (Pando-Barros Blancos). Marcos Paz and Pando-Barros Blancos are small cities with 54,000 and 58,000 residents, respectively; Bariloche (Argentina) and Temuco (Chile) are larger cities with 134,000 and 245,000 residents, respectively. Urban population was included in those sites. These study locations were selected based on population characteristics reflecting country averages.

A 4-stage stratified sampling method was used to select a representative sample of the population. In the first stage, census radii were randomly selected from each of the four locations, stratified by socio-economic level. In the second stage, a number of blocks proportional to the radius size were randomly selected. In the third stage, households within each block were selected by systematic random sampling. All members between 35–74 years in the selected households were listed to create the study sampling frame. In the final stage of sampling, one listed member per household was randomly selected to be included in the study.

The study protocol was approved by institutional review boards and ethics committees of all participating institutions in Argentina, Chile, Uruguay and the US: Comité de Ética de Protocolos de Investigación del Hospital Italiano de Buenos Aires, Argentina, Comité Ética Científica Araucanía Sur, Temuco, Chile, Comité de Ética para Proyectos de Investigación—Facultad de Medicina—Universidad de la República Montevideo, Uruguay and Tulane University Human Research Protection Office. All study participants provided written informed consent.

### Data collection

Study data were collected at a home visit and a clinical visit. During the home survey, information was obtained on demographic characteristics, including age, sex, education, occupation, household income, and healthcare access; personal history of CVD and risk factors, physical activity using the International Physical Activity Questionnaire–Short Form [20]; and diet using a semi-quantitative, self-administered food frequency questionnaire adapted from the NCI Dietary History Questionnaire and validated in Argentina, Chile, and Uruguay [21]. Anthropometric measurements were obtained by trained and certified observers using standard protocols and techniques. Three blood pressure (BP) measurements were obtained with the participant in the seated position after 5 min of rest using a standard mercury or aneroid sphygmomanometer, and the mean of three readings was used for analysis. Body weight, height, and waist circumference were measured twice during the examination and the average of two measurements was used in all analyses. Weight was measured in light indoor clothing without shoes in kilograms to one decimal place, using standing scales. Height was measured without shoes in centimeters to one decimal place using a wall-mounted stadiometer. Waist circumference was measured at 1 cm above the navel at minimal respiration, in centimeters to one decimal place.

Overnight fasting blood specimens were obtained for measurement of lipids, creatinine, and glucose. The duration of fasting was verified before the blood specimen was obtained. Participants who had not fasted for at least 10 hours did not have their blood drawn. Blood glucose, total and HDL-cholesterol, triglycerides, and creatinine were measured using standard methods. Concentration of LDL-cholesterol was calculated using the Friedewald equation for participants who had < 400 mg/dL triglycerides [22].

### Definitions

Obesity was defined as body-mass index (BMI) ≥30 kg/m^2^ and overweight as BMI ≥25 and <30 kg/m^2^. Central obesity was defined as waist circumference ≥102 cm for men or ≥88 cm for women [23]. Low physical activity was defined as less than 600 MET-minutes/week of total physical activity which includes walking, and moderate- and vigorous-intensity activities. Low fruit and vegetable intake was defined as <5 servings per day. Hypertension was defined as mean systolic BP ≥140 mm Hg, and/or mean diastolic BP ≥90 mm Hg, and/or current use of antihypertensive medications. Dyslipidemia was defined as total cholesterol ≥240 mg/dL and/or LDL-cholesterol ≥160 mg/dL and/or HDL-cholesterol <40 mg/dL and/or triglyceride ≥200 mg/dL and/or use of lipid-lowering medication. Diabetes was defined as fasting glucose ≥126 mg/dL and/or self- reported history of diabetes and/or current use of insulin or antidiabetic medications. [24].

### Statistical analysis

The CESCAS I study was designed to provide precise estimates of the prevalence of CVD risk factors by sex and location in four age groups: 35–44, 45–54, 55–64 and 65–74 years old. All calculations were weighted to represent the general adult population aged 35–74 years in the study sites. Mean level of anthropometric obesity measurements and overweight and obesity prevalence estimates were calculated for the overall population and by the four age groups. Additionally, age-standardized prevalence estimates were calculated for men and women according to the overall 2010 population distribution in the Southern Cone of Latin America [25]. The association of overweight, obesity and central obesity with risk factor prevalence was assessed using crude prevalence odds ratios (OR) for men and women and adjusted OR which were adjusted for age, gender, education, site, current smoking, physical activity, hypertension, diabetes, and dyslipidemia, where appropriate. All data analyses were conducted using SAS 9.3 (SAS Institute, Cary NC) and Stata 13.0 (StataCorp, College Station, TX).

## Results

Of the 10,254 individuals randomly selected, 550 were never found at their homes and 1394 refused to participate. Of those 8310 who completed the home surveys, 786 did not attend the clinical examination. Thus, the final sample for this analysis includes 7524 participants (3165 men and 4359 women).

Mean BMI and waist circumference were 28.9 kg/m^2^ and 96.5 cm, respectively, among adults aged 35–74 years in the Southern Cone (Table 1). Women had higher BMI while men had higher waist circumference. BMI and waist circumference varied among cities. The overall prevalence of overweight, obesity, and central obesity were 41.3%, 35.7%, and 52.9%, respectively. The prevalence of obesity and central obesity were higher in women while overweight was higher in men (Table 2). Obesity, overweight and central obesity increased with age. Central obesity prevalence increased from 29.7% (95% Confidence Interval [CI]: 26.0 to 33.4) in men aged 35–44 years to 44.3% (95%CI: 40.3 to 48.3) in those between 65 to 74 years. In the same age ranges, in women central obesity prevalence increased from 61.2% (95%CI: 57.6, 64.8) to 80.0% (95%CI: 77.1 10 82.9). Approximately 52.3% of individuals did not graduate from secondary school. In women, obesity and central obesity were less common among those with education levels including secondary school and university with an OR of 0.71 (95%CI: 0.59 to 0.86) and 0.42 (95%CI: 0.34 to 0.53), respectively (Tables 3 and 4). However, no association was observed between education and overweight, obesity and central obesity in men.

**Table 1:** Characteristics of study participants among men and women aged 35–74 years in the Southern Cone of Latin America.

	Overall	Men	Women
**No.**	7524	3165	4359
**Age Group**, n (%)			
35–44 yrs	1716 (22.8)	706 (22.3)	1010 (23.2)
45–54 yrs	2072 (27.5)	832 (26.3)	1240 (28.5)
55–64 yrs	2114 (28.1)	935 (29.5)	1179 (27.1)
65–74 yrs	1622 (21.6)	692 (21.9)	930 (21.3)
**Location**, n (%)			
Marcos Paz, Argentina	1991 (26.5)	792 (25.0)	1199 (27.5)
Bariloche, Argentina	1999 (26.6)	798 (25.2)	1201 (27.6)
Temuco, Chile	1950 (25.9)	923 (29.2)	1027 (23.6)
Pando-Barros Blancos, Uruguay	1584 (21.1)	652 (20.6)	932 (21.4)
**Education level**, n (%)			
Primary School	3409 (45.4)	1381 (43.7)	2028 (46.6)
Secondary School	2790 (37.1)	1200 (37.9)	1590 (36.5)
University	1318 (17.5)	582 (18.4)	736 (16.9)
Fasting Plasma Glucose	98.0 (97.3, 98.8)	99.8 (98.7, 100.8)	96.5 (95.4, 97.6)
Current Smoking	29.7 (28.4, 31.0)	33.3 (31.3, 35.3)	26.5 (24.8, 28.3)
SBP	127.1 (126.6, 127.6)	129.8 (129.1, 130.5)	124.6 (123.9, 125.3)
DBP	82.3 (82.0, 82.7)	84.7 (84.2, 85.1)	80.2 (79.8, 80.7)
Low Intake of fruit and vegetables	85.5 (84.6, 86.5)	89.8 (88.6, 91.0)	81.7 (80.3, 83.1)
Low Physical activity	35.2 (33.8, 36.5)	28.3 (26.4, 30.2)	41.3 (39.4, 43.1)
Body-mass index (kg/m^2^)	28.9 (28.7, 29.0)	28.5 (28.3, 28.7)	29.2 (29.0, 29.4)
Waist circumference (cm)	96.5 (96.1, 96.8)	98.8 (98.3, 99.3)	94.4 (93.9, 94.9)

Values are percentage or mean (95% confidence interval).

**Table 2:** Estimated prevalence of overweight, obesity and abdominal obesity among adults aged 35–74 years in the Southern Cone of Latin America.

	Overweight	Obesity	Central Obesity
Overall	41.3 (39.9, 42.7)	35.7 (34.4, 37.0)	52.9 (51.6, 54.3)
Men	47.7 (45.6, 49.8)	31.9 (30.0, 33.8)	35.7 (33.7, 37.7)
Age groups, years			
35–44	48.1 (44.0, 52.1)	29.6 (25.9, 33.3)	29.7 (26.0, 33.4)
45–54	48.7 (44.9, 52.4)	32.8 (29.4, 36.3)	35.4 (31.9, 38.9)
55–64	46.6 (43.1, 50.1)	34.7 (31.4, 38.0)	42.4 (39.0, 45.9)
65–74	46.2 (42.2, 50.2)	32.4 (28.6, 36.1)	44.3 (40.3, 48.3)
Women	35.5 (33.7, 37.3)	39.1 (37.3, 40.9)	68.4 (66.6, 70.1)
Age groups, years			
35–44	35.0 (31.4, 38.7)	35.0 (31.4, 38.6)	61.2 (57.6, 64.8)
45–54	36.3 (33.1, 39.5)	37.6 (34.4, 40.8)	66.3 (63.2, 69.3)
55–64	36.3 (33.1, 39.6)	43.5 (40.2, 46.8)	76.2 (73.5, 79.0)
65–74	34.1 (30.6, 37.6)	46.8 (43.2, 50.4)	80.0 (77.1, 82.9)
Education level			
Primary School	38.4 (36.3, 40.4)	40.9 (38.9, 42.9)	57.2 (55.2, 59.3)
Secondary School	42.9 (40.6, 45.1)	35.1 (33.0, 37.3)	52.7 (50.5, 54.9)
University	42.7 (39.5, 45.9)	29.4 (26.4, 32.3)	47.3 (44.1, 50.5)
Site			
Marcos Paz, Argentina	34.2 (31.9, 36.6)	44.7 (42.3, 47.2)	54.4 (51.9, 56.8)
Bariloche, Argentina	40.0 (37.7, 42.4)	32.2 (30.0, 34.4)	46.5 (44.1, 48.8)
Temuco, Chile	45.5 (43.0, 47.9)	35.6 (33.3, 37.9)	54.9 (52.5, 57.3)
Barros Blancos, Uruguay	35.1 (32.6, 37.6)	36.7 (34.2, 39.2)	57.6 (55.0, 60.2)

Data are weighted percentages (95% confidence intervals).

**Table 3:** Estimated prevalence of risk factors by BMI and Central Obesity among adults aged 35–74 years in the Southern Cone of Latin America.

	Body-mass index Intervals				Central Obesity	
	≤24.9 kg/m^2^	25.0–29.9 kg/m^2^	30.0–34.9 kg/m^2^	≥35.0 kg/m^2^	No	Yes
Men						
Less than Secondary School	36.5 (32.4, 40.7)	29.5 (26.9, 32.1)	34.9 (31.2, 38.7)	36.1 (29.5, 42.7)	32.2 (29.9, 34.5)	34.2 (31.3, 37.1)
Unemployed	4.9 (2.9, 6.9)	5.1 (3.7, 6.6)	4.9 (3.0, 6.8)	6.4 (2.7, 10.2)	4.9 (3.8, 6.1)	5.5 (3.9, 7.2)
Current Cigarette Smoking	46.5 (42.0, 51.0)	31.9 (29.0, 34.8)	28.2 (24.3, 32.1)	23.1 (16.8, 29.5)	36.5 (33.9, 39.0)	27.5 (24.4, 30.6)
Low Physical Activity	23.7 (19.8, 27.5)	27.2 (24.4, 29.9)	31.2 (27.2, 35.1)	38.0 (31.0, 45.1)	25.3 (22.9, 27.6)	34.0 (30.7, 37.2)
Low Intake of Fruit and Veg	90.3 (87.8, 92.9)	89.3 (87.5, 91.0)	90.6 (88.4, 92.9)	89.0 (84.7, 93.3)	89.7 (88.3, 91.2)	89.9 (88.0, 91.8)
Hypertension	25.2 (21.6, 28.7)	41.5 (38.6, 44.5)	58.4 (54.2, 62.6)	72.5 (65.9, 79.1)	34.7 (32.3, 37.2)	62.4 (59.1, 65.7)
Diabetes	5.4 (3.6, 7.2)	8.8 (7.2, 10.3)	14.9 (12.1, 17.7)	22.4 (16.6, 28.1)	6.7 (5.5, 7.8)	17.6 (15.2, 20.1)
Dyslipidemia	51.3 (46.7, 55.9)	69.9 (67.0, 72.7)	75.3 (71.7, 78.9)	81.6 (75.9, 87.3)	63.4 (60.8, 66.0)	77.1 (74.4, 79.8)
CKD	1.0 (0.3, 1.7)	2.0 (1.4, 2.6)	2.3 (1.4, 3.2)	1.7 (0.3, 3.0)	1.4 (0.9, 1.8)	2.7 (1.8, 3.5)
Individuals with ≥3 RF*	46.3 (41.8, 50.8)	56.6 (53.5, 59.6)	68.9 (65.0, 72.8)	72.3 (65.7, 78.9)	52.1 (49.4, 54.7)	70.5 (67.4, 73.5)
Individuals with ≥5 RF	2.2 (1.1, 3.4)	4.6 (3.4, 5.8)	5.9 (4.1, 7.7)	14.2 (9.1, 19.4)	3.2 (2.4, 4.0)	8.7 (6.8, 10.5)
Women						
Less than Secondary School	26.5 (23.6, 29.4)	34.1 (31.2, 37.0)	37.3 (33.9, 40.8)	48.5 (43.8, 53.1)	28.8 (26.0, 31.6)	38.1 (36.0, 40.2)
Unemployed	7.8 (5.4, 10.2)	6.2 (4.6, 7.9)	6.9 (4.7, 9.0)	6.3 (3.7, 8.8)	7.1 (5.2, 9.0)	6.7 (5.4, 7.9)
Current Cigarette Smoking	35.1 (31.6, 38.6)	27.3 (24.3, 30.3)	20.8 (17.5, 24.0)	19.7 (15.7, 23.7)	33.4 (30.2, 36.6)	23.4 (21.4, 25.5)
Low Physical Activity	36.1 (32.4, 39.7)	39.7 (36.4, 42.9)	44.4 (40.6, 48.1)	48.7 (44.0, 53.4)	35.1 (31.8, 38.4)	44.2 (41.9, 46.4)
Low Intake of Fruit and Veg	82.3 (79.6, 85.0)	81.1 (78.7, 83.6)	79.8 (76.7, 82.8)	85.0 (81.9, 88.2)	83.0 (80.7, 85.4)	81.1 (79.3, 82.8)
Hypertension	21.0 (18.3, 23.7)	35.1 (32.2, 38.0)	45.7 (42.0, 49.5)	56.0 (51.2, 60.8)	22.6 (20.1, 25.2)	44.0 (41.8, 46.3)
Diabetes	4.7 (3.3, 6.0)	11.5 (9.5, 13.5)	18.3 (15.4, 21.2)	28.4 (24.2, 32.6)	4.8 (3.4, 6.2)	18.3 (16.6, 20.0)
Dyslipidemia	35.8 (32.3, 39.4)	50.5 (47.3, 53.8)	55.1 (51.3, 58.9)	61.2 (56.6, 65.8)	36.0 (32.8, 39.2)	55.8 (53.6, 58.1)
CKD	1.3 (0.7, 1.9)	1.5 (0.9, 2.1)	3.1 (2.1, 4.2)	3.5 (2.0, 4.9)	1.1 (0.6, 1.6)	2.7 (2.1, 3.3)
Individuals with ≥3 RF	36.2 (32.7, 39.7)	48.0 (44.7, 51.2)	53.1 (49.3, 56.9)	66.5 (62.1, 70.9)	36.6 (33.4, 39.8)	54.9 (52.6, 57.1)
Individuals with ≥5 RF	2.1 (1.2, 3.0)	3.2 (2.1, 4.4)	7.2 (5.3, 9.1)	10.4 (7.9, 12.9)	1.9 (1.0, 2.9)	6.5 (5.4, 7.5)

Data are weighted percentages (95% confidence intervals).

* RF: risk factors (including low intake of fruit and vegetables, low physical activity, current cigarette smoking, hypertension, chronic kidney disease, dyslipidemia, and diabetes).

**Table 4:** Univariate odds ratios (ORs) and 95% confidence intervals (95% CIs) for the association of risk factors with Obesity and Central Obesity among adults aged 35–74 years in the Southern Cone of Latin America.

Risk factors	Obesity	Central Obesity
	OR (95% CI)	OR (95% CI)
	**Male**	
Age	1.006(0.998, 1.014)	1.023(1.012, 1.031)
Education level		
Secondary School vs. Primary	0.85(0.70, 1.03)	0.89(0.74, 1.07)
University vs. Primary	0.86(0.67, 1.09)	0.96(0.76, 1.21)
Current Smoking	0.65(0.53, 0.80)	0.66(0.55, 0.80)
Low Physical activity	1.38(1.14, 1.68)	1.52(1.26, 1.84)
Low Intake of fruit and vegetables	1.08(0.82, 1.41)	1.02(0.78, 1.32)
Hypertension	2.79(2.32, 3.36)	3.12(2.61, 3.73)
Diabetes	2.38(1.86, 3.05)	3.00(2.34, 3.84)
Dyslipidemia	1.83(1.50, 2.24)	1.94(1.61, 2.35)
Chronic kidney disease	1.28(0.80, 2.04)	1.93(1.23, 3.04)
	**Women**	
Age	1.016 (1.009, 1.023)	1.033(1.025, 1.041)
Education level		
Secondary School vs. Primary	0.75(0.63, 0.88)	0.8.0(0.67, 0.96)
University vs. Primary	0.45(0.36, 0.56)	0.48(0.39, 0.59)
Current Smoking	0.58(0.48, 0.70)	0.61(0.51, 0.73)
Low Physical activity	1.38(1.18, 1.62)	1.46(1.23, 1.74)
Low Intake of fruit and vegetables	1.01(0.84, 1.23)	0.88(0.71, 1.07)
Hypertension	2.40(2.05, 2.80)	2.69(2.27, 3.19)
Diabetes	3.01(2.42, 3.74)	4.45(3.20, 6.18)
Dyslipidemia	1.69(1.45, 1.98)	2.24(1.90, 2.65)
Chronic kidney disease	2.31(1.54, 3.46)	2.50(1.48, 4.20)

In the Southern Cone, 85.5% of adults (89.8% of men and 81.7% of women) aged 35–74 years ate less than five servings of fruit or vegetables per day. The low intake of fruit and vegetables was consistent across cities and age groups. However, obesity measurements were not associated with low intake of fruit and vegetables (Tables 3 and 4). About 35.2% of adults (28.3% of men and 41.3% of women) had low physical activity in the Southern Cone, which varied by city and increased with age. Obesity and central obesity were associated with low physical activity in men with an OR of 1.34 (95%CI: 1.06 to 1.71) and 1.52 (95%CI: 1.26 to 1.84), respectively and in women with an OR of 1.34 (95%CI: 1.12 to1.60) and 1.52 (95%CI: 1.26 to1.84), respectively (Table 4). In addition, 29.7% of adults (33.3% of men and 26.5% of women) in the Southern Cone were current cigarette smokers. The prevalence of cigarette smoking was consistent among the four study cities and decreased with age. Obesity and central obesity was inversely associated with current smoking in men with an OR of 0.65 (95%CI: 0.53 to 0.80) and 0.66 (95%CI: 0.55 to 0.80) and in women with an OR of 0.58 (95%CI: 0.48 to 0.70) and 0.61 (95%CI: 0.51 to 0.73), respectively.

After adjusting for age, gender, education, site, current smoking, physical activity, hypertension, diabetes, and dyslipidemia, in men obesity and central obesity were associated with an increased prevalence of diabetes (OR 2.38 and 3.00), hypertension (OR 2.79 and 3.12) and dyslipidemia (OR 1.83 and 1.94). In women, obesity and central obesity were also associated with an increased prevalence of diabetes (OR 3.01 and 4.45), hypertension (OR 2.4 and 2.69), and dyslipidemia (OR 1.69 and 2.4) (Table 5). The prevalence of hypertension increases dramatically from 25.2% in those with normal BMI to 72.5% in those with BMI > 35 kg/m^2^. In the same groups, diabetes mellitus prevalence increased from 5.4% to 22.4%, and dyslipidemia prevalence from 51.3% to 81.6%. Obesity was associated with a higher proportion of individuals with multiple CVD risk factors, including low intake of fruit and vegetables, low physical activity, current cigarette smoking, hypertension, chronic kidney disease, dyslipidemia, and diabetes. The proportions of individuals with 3 or more risk factors were 46.3% in individuals who were normal weight and 68.9% in those who were obese. The proportions of individuals with 5 or more risk factors were 2.2% in individuals who were normal weight and 14.2% in those who were obese (Table 3).

**Table 5:** Multivariate odds ratios (ORs) and 95% confidence intervals (95% CIs) for the association of risk factors with Obesity and Central Obesity among adults aged 35–74 years in the Southern Cone of Latin America.

Risk factors	Obesity	Central Obesity
	OR (95% CI)	OR (95% CI)
	**Male**	
Current Smoking	0.76(0.61, 0.94)	0.79(0.64, 0.96)
Low Physical activity	1.29(1.05, 1.59)	1.44(1.17, 1.76)
Hypertension	2.50(2.07, 3.03)	2.77(2.30, 3.33)
Diabetes	1.84(1.41, 2.39)	2.29(1.77, 2.96)
Dyslipidemia	1.69(1.38, 2.08)	1.77(1.45, 2.17)
	**Women**	
Education level		
Secondary School vs. Primary	0.91(0.76, 1.09)	0.98(0.81, 1.18)
University vs. Primary	0.58(0.46, 0.73)	0.63(0.50, 0.79)
Current Smoking	0.64(0.53, 0.79)	0.67(0.55, 0.81)
Low Physical activity	1.34(1.14, 1.59)	1.41(1.17, 1.70)
Hypertension	1.86(1.57, 2.19)	2.07(1.72, 2.49)
Diabetes	2.33(1.84, 2.95)	3.38(2.37, 4.82)
Dyslipidemia	1.41(1.20, 1.67)	1.89(1.59, 2.26)

Adjusted for age, gender, education, site, current smoking, physical activity, hypertension, diabetes and dyslipidemia. Only significant factors are shown in the table.

## Discussion

The most striking findings in our study are the very high prevalence of overweight, obesity, and central obesity in the study population and the strong association of obesity with CVD risk factors. Overweight and obesity affected 3 out of 4 adults and central obesity affected half of adults in the Southern Cone of Latin America. Obesity is an important risk factor for hypertension, dyslipidemia, diabetes, and metabolic syndrome, which were all highly prevalent in the study population.

Prevalence of obesity has reached alarming levels in some countries. In the United States, an overweight and obese population of 68.5% has been reported in adults > 20 years [8]. Population growth and social development in Latin America has led to a demographic transition, increasing the frequency of obesity occurrence, which greatly differs among the countries of the region [5,9,10]. Over the last few decades, the Brazilian population has increased consumption of processed foods, with a rise in fat intake, specifically saturated fat, along with increased sodium and sugar intake [11]. This is likely a factor contributing to the increased obesity prevalence seen in that population—14.8% in the population >20 years old in 2010—with the greatest increase occurring in both the lowest income quintile and in less-educated women [11]. In Mexico, the 2012 National Health Survey (NHS) revealed alarming figures, with a prevalence of overweight and obesity of 64.9% in men and 73% in women >20 years old [12]. In Colombia in 2007, an estimated 32.2% of the population were overweight and 13.7% were obese between 18 and 69 years of age [13]. In Argentina, in a survey using self-reported data, 37.1% of respondents were overweight and 20.8% were obese among those older than 17 years, with an increase directly related to aging [14]. In Chile, prevalence rates of obesity reached 25.1% in persons older than 14 years, and they were higher in women, with a rate of 30.7% [15]. In the Uruguay National Survey the combined prevalence of overweight and obesity was 64.7% [16]. All of these prevalences are influenced by national age distributions and are not standardized, which limits comparisons. In several countries, the pattern of obesity occurring more frequently in both lower socioeconomic levels and lower levels of education is replicated.

The Cardiovascular Risk Factor Multiple Evaluation in Latin America (CARMELA) study examined CVD risk factors among individuals living in seven major cities in Latin American countries, including Buenos Aires and Santiago, the capitals of Argentina and Chile, respectively [26]. As compared to CARMELA, our study reported a higher prevalence of hypertension, diabetes and obesity. If the comparisons are restricted to only Buenos Aires and Santiago, these differences are more striking. The differences in CVD risk factors between CARMELA and ours might partially reflect the secular increases in CVD risk factors in the Southern Cone, since measurements in CARMELA were performed in 2005, as compared to ours in 2011–12. A high prevalence of obesity and CVD risk factors were reported in Latinos living in the US. The Hispanic Community Health Study/Study of Latinos (HCHS/SOL) reported 36.5% of male and 42.6% of female Latinos aged 18–74 years in the US were obese [27].

Body fat distribution is also an important risk factor for obesity-related health problems. Several studies have shown that the presence of central obesity has been linked to coronary artery disease [28, 29, 30]. Even in coronary patients with normal BMI, the presence of central obesity is directly associated with increased mortality [30–32]. Factors related to the development of acute myocardial infarction (AMI) were examined in Latin America as well as worldwide by the INTERHEART (A Study of Risk Factors for First Myocardial Infarction in 52 Countries and Over 27,000 Subjects) [17,28]. This study analyzed 15,152 cases of first AMI and 14,820 control subjects from 52 countries, of which 1,237 cases and 1,888 control subjects were from 6 Latin American countries (Argentina, Brazil, Colombia, Chile, Guatemala, and Mexico). In the Latin American participants in INTERHEART study, an increased waist/hip ratio was associated with an increased risk of AMI after adjusting for other risk factors, with an overall OR of 2.5 (95%CI: 2.0 to 3.14) and a higher OR of 4.1 (95%CI: 2.6 to 6.5) in women. Increased waist/hip ratio was the most prevalent risk factor for AMI in this population. This association was also higher than that found for other INTERHEART regions studied [17].

Our study is one of the first studies in Latin America to systematically and objectively examine CVD risk factors in a representative sample of a general population. Physical and biochemical risk factors were measured using standard methods, which can reduce potential bias due to self-reported data. In addition, a stringent quality control procedure was implemented in all stages of the study. This study provides the most recent and reliable data on CVD risk factors in the general adult population in the Southern Cone of Latin America and responds to the World Heart Organization request in its Global Action Plan for the prevention and control of non-communicable diseases for international studies of overweight and obesity prevalence [33]. The major limitation of our study is its cross sectional design which precludes causal inference between risk factors and obesity, Also we did not made adjustments for history of cardiovascular disease or diet considering that they can be in the causal pathway. Then, our findings of association of obesity with cardiovascular risk factors should be interpreted cautiously.

In summary, our study indicates that CVD risk factors are highly prevalent in the general population in the Southern Cone of Latin America and strongly related with obesity. The prevalence of CVD risk factors in this population is higher than in other LMICs as well as in high-income countries [30, 31] and our results suggest that this increased prevalence is related with the high prevalence of obesity in our communities. Cardiovascular disease are the main cause of mortality and disability in the southern cone of America and it is unlikely that they will be limited unless the current obesity epidemic is controlled. Our data suggest that national efforts toward the prevention, treatment, and control of CVD risk factors should be a public health priority in the Southern Cone of Latin America.

## Supporting Information

S1 File Study database. (XLSX)

## References

[1] de Fatima Marinho de Souza M, Gawryszewski VP, Orduñez P, Sanhueza A, Espinal MA. Cardiovascular disease mortality in the Americas: current trends and disparities. Heart. 2012; 98:1207–12 10.1136/heartjnl-2012-301828 22826558

[2] Lanas F, Serón P, Lanas A. Cardiovascular Disease in Latin America: The Growing Epidemic. Prog Cv Dis 2014; 57 (3): 262–268 10.1016/j.pcad.2014.07.007 25443823

[3] Murray CJ, Lopez AD. The global burden of disease: a comprehensive assessment of mortality and disability from diseases, injuries and risk factors in 1990 and projected to 2020. Cambridge, MA: Harvard University Press, 1996.

[4] Yusuf S, Reddy S, Ounpuu S, Anand S. Global burden of cardiovascular diseases: part I: general considerations, the epidemiologic transition, risk factors, and impact of urbanization. Circulation. 2001; 104:2746–53. 11723030 10.1161/hc4601.099487

[5] World Health Organization. Obesity: Preventing and managing the global epidemic. WHO Technical report series N 894. 2000, Geneva 11234459

[6] Eckel RH, York DA, Rossner S, Hubbard V, Caterson I, St Jeor ST, et al American Heart Association. Prevention Conference VII: obesity a Worldwide epidemic related to heart disease and stroke: executive summary. Circulation. 2004; 110: 2968–2975. 10.1161/01.CIR.0000140086.88453.9A 15520336

[7] Logue J, Murray HM, Welsh P, Shepherd J, Packard C, Macfarlane P, et al Obesity is associated with fatal coronary heart disease independently of traditional risk factors and deprivation. Heart. 2011;97:564–8 10.1136/hrt.2010.211201 21324888

[8] Ogden CL, Carroll MD, Kit BK, Flegal KM. Prevalence of childhood and adult obesity in the United States, 2011–2012. JAMA. 2014 2 26;311(8):806–14 10.1001/jama.2014.732 24570244 PMC4770258

[9] Uauy R, Albala C, Kain J. Obesity trends in Latin America: transiting form under-to overweight. J Nutr. 2001; 131: 893S–899S 11238781 10.1093/jn/131.3.893S

[10] Miranda JJ, Herrera VM, Chirinos JA, Gómez LF, Perel P, Pichardo R et al Major cardiovascular risk factors in Latin America: a comparison with the United States. The Latin American Consortium of Studies in Obesity [LASO]. PLOS ONE. 2013;8:e54056 10.1371/journal.pone.0054056 23349785 PMC3547948

[11] Schmidt MI, Duncan BB, Azevedo e Silva G, Menezes AM, Monteiro CA, Barreto SM et al Chronic non-communicable diseases in Brazil: burden and current challenges. Lancet. 2011; 377:1949–61 10.1016/S0140-6736(11)60135-9 21561658

[12] Encuesta Nacional de Salud y Nutrición 2012. Available: http://ensanut.insp.mx/informes/ENSANUT2012ResultadosNacionales.pdf. Accessed 13 December 2015

[13] Encuesta Nacional de Salud ENS 2007 Available: http://www.scp.com.co/ArchivosSCP/boletines_Pedianet/DocumentosPedianet/Encuesta_Nacional_de_Salud_2007.pdf. Accessed 13 December 2013

[14] Encuesta Nacional de Factores de Riesgo. Argentina 2013. Available: http://msal.gov.ar/ENT/VIG/Publicaciones/Encuestas_Poblacionales/PDF/Encuesta%20Nacional%20De%20Factores%20De%20Riesgo%202005%20-%20Version%20Breve.pdf. Accessed 13 December 2015

[15] Encuesta Nacional de Salud ENS Chile 2009–2010. Available: http://www.minsal.cl/portal/url/item/bcb03d7bc28b64dfe040010165012d23.pdf. Accessed 13 December 2015

[16] National Risk Factor Survey, Uruguay 2013. Ministerio de Salud de Uruguay. Preliminary results. Available: http://www.msp.gub.uy/noticia/presentaci%C3%B3n-de-resultados-2%C2%AA-encuesta-nacional-de-factores-de-riesgo-de-enfermedades. Accessed 13 December 2015

[17] Lanas F, Avezum A, Bautista LE, Diaz R, Luna M, Islam S, et al Risk factors for acute myocardial infarction in Latin America: the INTERHEART Latin American study. Circulation. 2007; 115(9):1067–74. 10.1161/CIRCULATIONAHA.106.633552 17339564

[18] Rubinstein AL, Irazola VE, Poggio R, Bazzano L, Calandrelli M, Lanas Zanetti FT, et al Detection and follow-up of cardiovascular disease and risk factors in the Southern Cone of Latin America: the CESCAS I study. BMJ Open. 2011 5 26;1(1):e000126 10.1136/bmjopen-2011-000126 22021769 PMC3191438

[19] Rubinstein AL, Irazola VE, Calandrelli M, Elorriaga N, Gutierrez L, Lanas F, et al Multiple cardiometabolic risk factors in the Southern Cone of Latin America: A population-based study in Argentina, Chile, and Uruguay Int J Cardiol. 2015 1 27;183C:82–88. 10.1016/j.ijcard.2015.01.062 PMC4382451 25662056

[20] Craig CL, Marshall AL, Sjöström M, Bauman AE, Booth ML, Ainsworth BE, et al International physical activity questionnaire: 12-country reliability and validity. Med Sci Sports Exerc. 2003; 35(8):1381–95. 10.1249/01.MSS.0000078924.61453.FB 12900694

[21] Elorriaga N, Irazola VE, Defago MD, Britz M, Martínez-Oakley SP, Witriw AM, et al Validation of a self-administered FFQ in adults in Argentina, Chile and Uruguay. Public Health Nutrition 2014 10.1017/S1368980013003431 24476763 PMC10271131

[22] Friedewald WT, Levy RI, Fredrickson DS. Estimation of the concentration of low-density lipoprotein cholesterol in plasma, without use of the preparative ultracentrifuge. Clin Chem. 1972; 18:499–502 4337382

[23] Executive Summary of The Third Report of The National Cholesterol Education Program (NCEP) Expert Panel on Detection, Evaluation, And Treatment of High Blood Cholesterol In Adults (Adult Treatment Panel III). JAMA 2001;285(19): 2486–97 11368702 10.1001/jama.285.19.2486

[24] American Diabetes Association. Diagnosis and classification of diabetes mellitus Diabetes Care. 2014; 37:S81–S90. 10.2337/dc14-S081 24357215

[25] Ahmad OB, Boschi-Pinto C, Lopez AD, Murray CJL, Lozano R, Inoue M. Age Standardization of Rates: a New WHO Standard. Geneva, Switzerland: Global Programme on Evidence for Health Policy, World Health Organization; 2000. GPE Discussion Paper No. 31).

[26] Hernández-Hernández R, Silva H, Velasco M, Pellegrini F, Macchia A, Escobedo J, et al Hypertension in seven Latin American cities: the Cardiovascular Risk Factor Multiple Evaluation in Latin America (CARMELA) study. J Hypertens. 2010; 28(1):24–34. 10.1097/HJH.0b013e328332c353 19809362

[27] Daviglus ML, Pirzada A, Talavera GA. Cardiovascular disease risk factors inthe Hispanic/Latino population: lessons from the Hispanic Community Health Study/Study of Latinos (HCHS/SOL). Prog Cardiovasc Dis. 2014; 5:230–6. 10.1016/j.pcad.2014.07.006 25242694

[28] Yusuf S, Hawken S, Ounpuu S, Dans T, Avezum A, Lanas F et al; INTERHEART Study Investigators. Effect of potentially modifiable risk factors associated with myocardial infarction in 52 countries (the INTERHEART study): case-control study. Lancet. 2004; 364:937–52. 10.1016/S0140-6736(04)17018-9 15364185

[29] Lee CD, Jacobs DR Jr, Schreiner PJ, Iribarren C, Hankinson A. Abdominal obesity and coronary artery calcification in young adults: the Coronary Artery Risk Development in Young Adults [CARDIA] Study. Am J Clin Nutr. 2007; 86:48–54. 17616762 10.1093/ajcn/86.1.48

[30] Gruson E, Montaye M, Kee F, Wagner A, Bingham A, Ruidavets JB et al Anthropometric assessment of abdominal obesity and coronary heart disease risk in men: the PRIME study. Heart. 2010;96:136–40. 10.1136/hrt.2009.171447 19561364

[31] Coutinho T, Goel K, Corrêa de Sá D, Kragelund C, Kanaya AM, Zeller M, et al Central obesity and survival in subjects with coronary artery disease: a systematic review of the literature and collaborative analysis with individual subject data. J Am Coll Cardiol. 2011;57:1877–86. 10.1016/j.jacc.2010.11.058 21545944

[32] Coutinho T, Goel K, Corrêa de Sá D, Carter RE, Hodge DO, Kragelund C, et al Combining body mass index with measures of central obesity in the assessment of mortality in subjects with coronary disease: role of "normal weight central obesity". J Am Coll Cardiol. 2013;61:553–60. 10.1016/j.jacc.2012.10.035 23369419

[33] World Health Organization. Global Action Plan for the Prevention and Control of Noncommunicable Diseases 2013–2020. Geneva: World Health Organization; 2013.

